# Fluorescent reporter assays provide direct, accurate, quantitative measurements of MGMT status in human cells

**DOI:** 10.1371/journal.pone.0208341

**Published:** 2019-02-27

**Authors:** Zachary D. Nagel, Andrew A. Beharry, Patrizia Mazzucato, Gaspar J. Kitange, Jann N. Sarkaria, Eric T. Kool, Leona D. Samson

**Affiliations:** 1 Department of Biological Engineering, Massachusetts Institute of Technology, Cambridge, Massachusetts, United States of America; 2 Department of Chemistry, Stanford University, Stanford, California, United States of America; 3 Department of Radiation Oncology, Mayo Clinic, Rochester, Minnesota, United States of America; Dana Farber Cancer Institute, UNITED STATES

## Abstract

The DNA repair protein *O*^6^-methylguanine DNA methyltransferase (MGMT) strongly influences the effectiveness of cancer treatment with chemotherapeutic alkylating agents, and MGMT status in cancer cells could potentially contribute to tailored therapies for individual patients. However, the promoter methylation and immunohistochemical assays presently used for measuring MGMT in clinical samples are indirect, cumbersome and sometimes do not accurately report MGMT activity. Here we directly compare the accuracy of 6 analytical methods, including two fluorescent reporter assays, against the *in vitro* MGMT activity assay that is considered the gold standard for measuring MGMT DNA repair capacity. We discuss the relative advantages of each method. Our data indicate that two recently developed fluorescence-based assays measure MGMT activity accurately and efficiently, and could provide a functional dimension to clinical efforts to identify patients who are likely to benefit from alkylating chemotherapy.

## Introduction

*O*^6^-methylguanine DNA Methyltransferase (MGMT; also known as alkylguanine alkyltransferase, AGT) repairs DNA damage induced by endogenous, environmental, and therapeutic alkylating agents, and is the most important pathway for repairing *O*^6^-alkylguanine adducts in human cells [1, 2]. MGMT prevents cell killing by repairing *O*^6^-methylguanine (*O*^6^-MeG) cytotoxic DNA lesions induced by the cancer chemotherapy agents Temozolomide and Dacarbazine, and cytotoxic *O*^6^-chloroethylguanine lesions induced by cross-linking agents such as BCNU [3]. These chemotherapeutic agents are used to treat a variety of cancers including glioblastoma, metastatic melanoma, and Hodgkins lymphoma. Clinical studies have demonstrated that glioblastoma patients with MGMT deficient tumors exhibit longer overall survival following treatment with temozolomide [4], and are more likely to respond to radiotherapy [5], highlighting the potential for personalized cancer therapy based on MGMT status in cancer cells.

Although MGMT diminishes the effectiveness of cancer therapies, it also plays a critical role protecting normal tissues from DNA damage. Over 10-fold inter-individual variation in MGMT activity has been observed in normal tissues [6, 7], and lower MGMT activity is associated with therapy related leukemia [8], myelotoxicity in patients receiving temozolomide [9], and lung cancer risk [10]. Since genetic variation [11–13], environmental exposures [7], and chemotherapy can each affect MGMT activity, methods that directly report MGMT function are best suited for studies measuring inter-individual differences in both normal tissues and cancer cells [14]. Studies investigating the relationships between MGMT activity and disease risk and cancer therapy outcomes have been limited by cumbersome and indirect assays that may not accurately predict MGMT activity.

Presently, MGMT status is assessed in clinical samples primarily using MGMT promoter methylation as a proxy for MGMT expression, and in some cases using immunohistochemistry; however, both methods can fail to reflect MGMT levels accurately. For example high levels of MGMT expression are possible in tumors with MGMT promoter hypermethylation due to expression of a previously unrecognized enhancer element [15]. Such observations highlight the need for functional assays that accurately measure MGMT activity to achieve personalized therapies based on DNA repair capacity assessments. We show here that two quantitative fluorescence-based assays including a small molecule reporter probe [16] and a plasmid based host cell reactivation assay [17, 18], accurately and efficiently measure MGMT activity in human cells. These assays are ready for use in preclinical studies, and have the potential to enable much-needed research aimed at tailoring cancer therapy to individual patients based on DNA repair capacity in tumor and normal tissues [19].

## Methods

### Cell lines

Seven B-lymphoblastoid cell lines including TK6 (RRID CVCL_0561) [20], TK6+MGMT [21], and five EBV transformed cell lines available from the Coriell Cell Repository were maintained in log phase in RPMI media with 20% FBS as previously described. The cell lines have been previously designated as follows: #4 (GM15223; CVCL_5W53), #5 (GM15245; CVCL_5W71), #12 (GM15385; CVCL_5Y77), #14 (GM15038; CVCL_5V37), and #16 (GM15072; CVCL_5V61) [22]; the same nomenclature is used here. Cells were obtained in 2001. The Coriell Cell Repository authenticates and tests cell cultures for contamination with mycoplasma, bacteria, and fungi; cells have not been authenticated by authors. Mycoplasma testing was carried out and found to be negative using a commercial PCR-based kit at the time cells were last passaged (November 2015).

### MGMT assays

Oligonucleotide cleavage assays were performed as described previously [23], and illustrated in (S1 Fig). To generate lysates, 1.5 x 10^7^ cells were collected, washed twice with PBS, and suspended in 400 μL of lysis buffer comprising 50 mM Tris, pH 7.5, 1 mM EDTA, 1 mM DTT, 5% glycerol, 50 mM NaCl, and 1 mM AEBSF (protease inhibitor). Cells were disrupted by sonication, cell debris was pelleted by centrifugation at 14,000g for 30 minutes at 4°C, supernatants were collected, and total protein concentration was determined using a BCA assay. Cell lysates were incubated at 37°C for 30 minutes with 4 pmoles of a ^32^P 5’-end labeled duplex comprising an oligonucleotide, GAACTXCAGCTCCGTGCTGGCCC, in which X represents *O*^6^MeG, and the corresponding complementary oligonucleotide. The reaction products were then purified by phenol/chloroform extraction and ethanol precipitation, dissolved, and finally digested using PstI restriction enzyme. *O*^6^MeG blocks PstI cleaveage, providing the basis for a gel-shift assay for the extent of MGMT-dependent lesion removal. Digests were analyzed by SDS PAGE followed by autoradiographic imaging, and densitometry was used to calculate the percentage of cleaved oligonucleotide. The linear range of the MGMT assay was established for each cell line by varying the amount cell lysate (between 10 and 400 μg) incubated with the duplex. MGMT activity in each cell lysate was calculated from the slope of a linear best fit of the percentage of oligonucleotide cleaved versus total protein concentration.

### Quantitative western blotting

Preparation of cell lysates and immunoblotting were performed as described previously [24]. Briefly, 25 μg of cell lysate (2.5 μg/μL in Laemli sample buffer) were separated by SDS-PAGE and transferred to a PVDF membrane. The membrane was blocked for 1 hour using Odyssey Blocking Buffer and incubated with a primary mouse antibody that binds human MGMT, followed by washing (4X) with PBS + 0.1% Tween-20 and 1 hour incubation with a secondary antibody, Licor IRDye 680RD donkey anti-mouse. After washing (4X) with PBS + 0.1% Tween-20, membranes were imaged in the 700 nm channel of an Odyssey imager. An actin antibody was used as a loading control. A representative gel and accompanying quantitation is available in S2 Fig.

### Quantitative real time PCR

qPCR data were described previously [17]. Total RNA was isolated using a Qiagen RNeasy kit, and mRNA was subsequently isolated using a Qiagen Oligotex kit according to the manufacturer’s protocols. Following DNase digest, cDNA was generated using poly-dT primers with reverse transcriptase. TaqMan qPCR was used to quantitate MGMT transcript levels relative to a GAPDH control. Primers and probes for MGMT (catalog number Hs.00172470) and GAPDH (Hs.99999905) were purchased from Applied Biosystems. A 20 μL reaction containing TaqMan Universal PCR Master Mix (Applied Biosystems), plus probes and cDNA was amplified by PCR using the following program: 10 minutes at 95°C, followed by 40 cycles of denaturing at 95°C for 15s followed by annealing and extension for 1 minute at 60°C.

### FM-HCR assays

FM-HCR assays have been published previously [17]. Briefly, reporter plasmids were generated by extension of *O*^6^MeG-containing oligonucleotides that were annealed to single-stranded plasmid DNA, followed by primer extension and ligation. The DNA lesion induces transcriptional errors that result in expression of functional mPlum fluorescent protein, unless repair removes *O*^6^MeG, the source of transcriptional errors. As a result, cells that efficiently repair *O*^6^MeG express relatively low levels of mPlum fluorescent protein, whereas MGMT deficient cells express relatively high levels of mPlum fluorescent protein. Transient transfection, flow cytometric analysis, and calculation of DNA repair capacity were described previously [17].

### Promoter methylation assays

Methylation specific PCR assays for promoter methylation were carried out as described previously [25]. Genomic DNA was extracted from 10^6^ cells from cell lines using a QIAamp DNA Kit, and bisulfite conversion of 1 microgram of the resulting gDNA was carried out using an EpiTect Bisulfite Kit (Qiagen). For PCR detection of unmethylated MGMT promoter sequences, the following primers were used: 5’TTTGTGTTTTGATGTTTGTAGGTTTTTGT-3’, 5’AACTCCACACTCTTCCAAAAACAAAACA-3’. For detection of methylated DNA, the following primers were used: 5’TTTCGACGTTCGTAGGTTTTCGC-3’, 5’GCACTCTTCCGAAAACGAAACG-3’. Approximately 100 ng of gDNA was combined with primers at a final concentration of 400 nM, and amplified with 1 unit of AmpliTaq Gold DNA polymerase for 35 cycles (annealing at 59 °C, and extension at 72 °C). PCR products were analyzed on a 3% agarose gel visualized with ethidium bromide (S3 Fig).

### Fluorogenic real-time reporter (NR-1) for repair by MGMT

Cell lysates were prepared from approximately 10^8^ cells using the procedure described above for MGMT assays. Cell lysates were analyzed using a recently reported DNA based fluorescent probe (NR-1) comprising a short DNA oligomer containing a fluorophore and an *O*^6^-benzylguanine nucleoside that is modified with a quencher dye [16]. Repair by MGMT separates the quencher from the fluorophore, leading to an increase in fluorescence. Cell lysates (800 μg total protein) were combined with 50 nM of NR-1 in a 96-well plate (final volume 200 μL), and incubated for 2 hours at 37°C. Fluorescence at 488 nm was measured with a plate reader. We observed that combining NR-1 with TK6 cell lysates or as purified BSA, both of which lack MGMT, leads to an approximately 2-fold increase in fluorescent signal, indicating that a relatively small but significant MGMT-independent increase in NR-1 fluorescence in the presence of proteins. Thus, to calculate MGMT activity, the fluorescent signal from MGMT deficient TK6 cell lysates combined with fluorescent probe was subtracted from the fluorescence values measured for all other cell lysates combined with fluorescent probe.

### Statistical analysis

For each method, error bars represent standard deviation from three biological replicates (carried out with materials independently prepared from the same cell line on different days). One-way ANOVA with Tukey’s multiple comparisons test was used to determine the ability of assays to distinguish MGMT levels or activity among the cell lines. All statistical analyses were carried out in Graphpad Version 7.0c.

## Results

**Fig 1:**
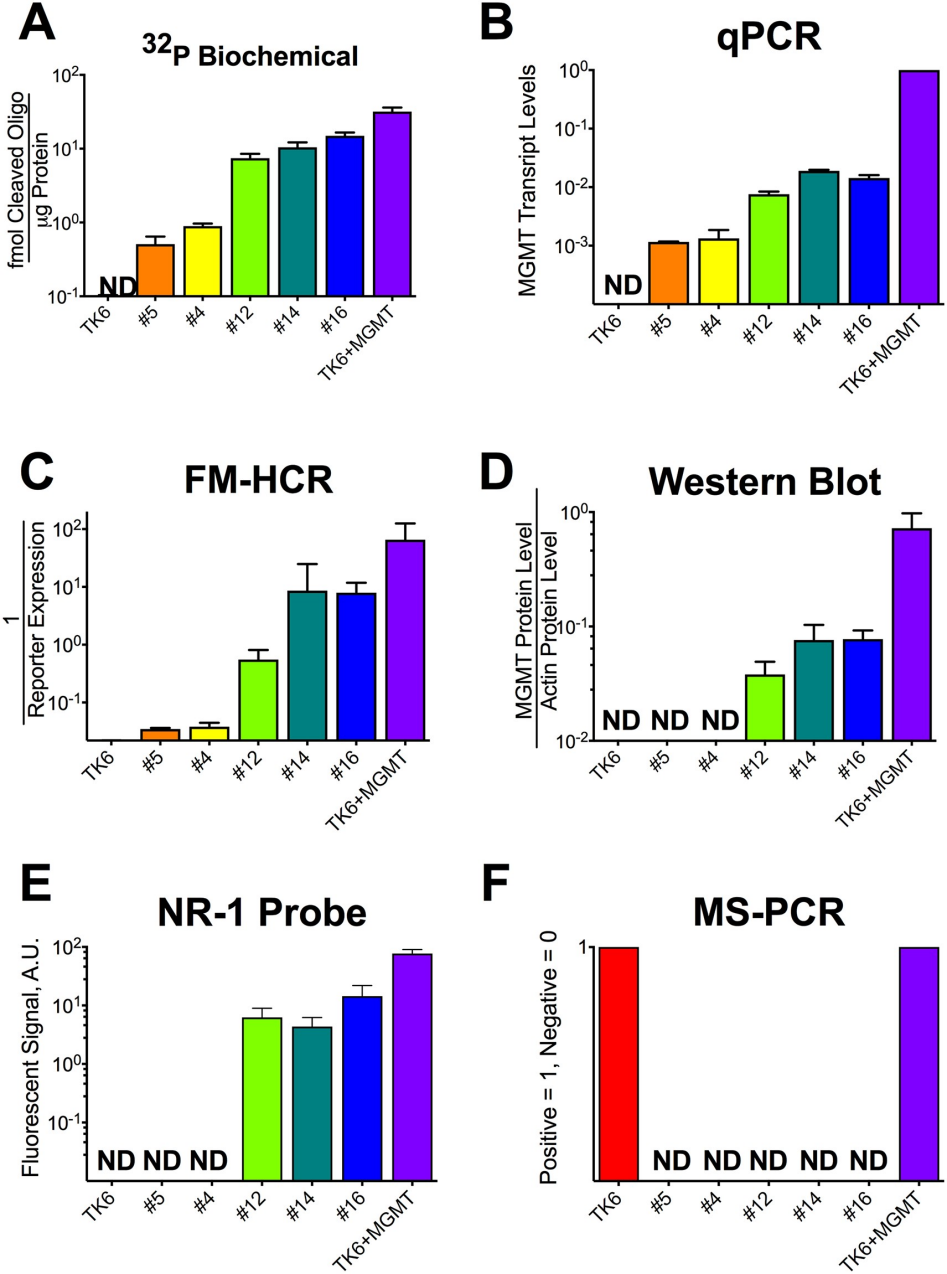
MGMT activity measured by 6 methods in 7 cell lines. A) MGMT activity reported as femtomoles of cleaved ^32^P labeled *O*^6^-MeG containing oligonucleotide per microgram of protein in cell lysates. B) MGMT transcript levels normalized to TK6+MGMT. C) MGMT activity measured by FM-HCR, reported as the inverse of reporter expression. D) MGMT protein levels in cell lysates measured by quantitative western blotting and normalized to GAPDH protein levels. E) MGMT activity in cell lysates measured using the NR-1 fluorescent probe and reported in arbitrary units of fluorescence. F) Results of methylation specific PCR assays for MGMT promoter methylation; a value of 1 was assigned to the three cell lines in which promoter methylation was detected. Cell lines were ranked in ascending order of MGMT activity measured by the biochemical assay in panel A; the order and color scheme is preserved in each panel. Error bars represent the standard deviation of at least 3 measurements, and “ND” indicates that the 95% confidence interval for the measured parameter included zero. Data have been log transformed for optimal data visualization.

A panel of 24 lymphoblastoid cell lines derived from apparently healthy individuals from diverse genetic backgrounds [26], Coriell #1–24, has been characterized previously for MGMT levels using transcriptional profiling and MGMT activity using a fluorescence based multiplex host cell reactivation (FM-HCR) assay [17]. Focusing on a subset of these cell lines (Coriell #4, #5, #12, #14 and #16), together with an MGMT-deficient negative control TK6, and a TK6-derived MGMT proficient positive control, TK6+MGMT [21], we have measured MGMT levels or activity in the seven cell lines using six different methods (Fig 1, S1 and S2 Tables). We chose to focus on this representative subset of cell lines because previous data from our laboratory revealed that they span the entire range of sensitivity to *O*^6^MeG generating alkylating agents observed previously for the larger set of cell lines [22], which can be explained in part by differences in MGMT activity [27].

**Fig 2:**
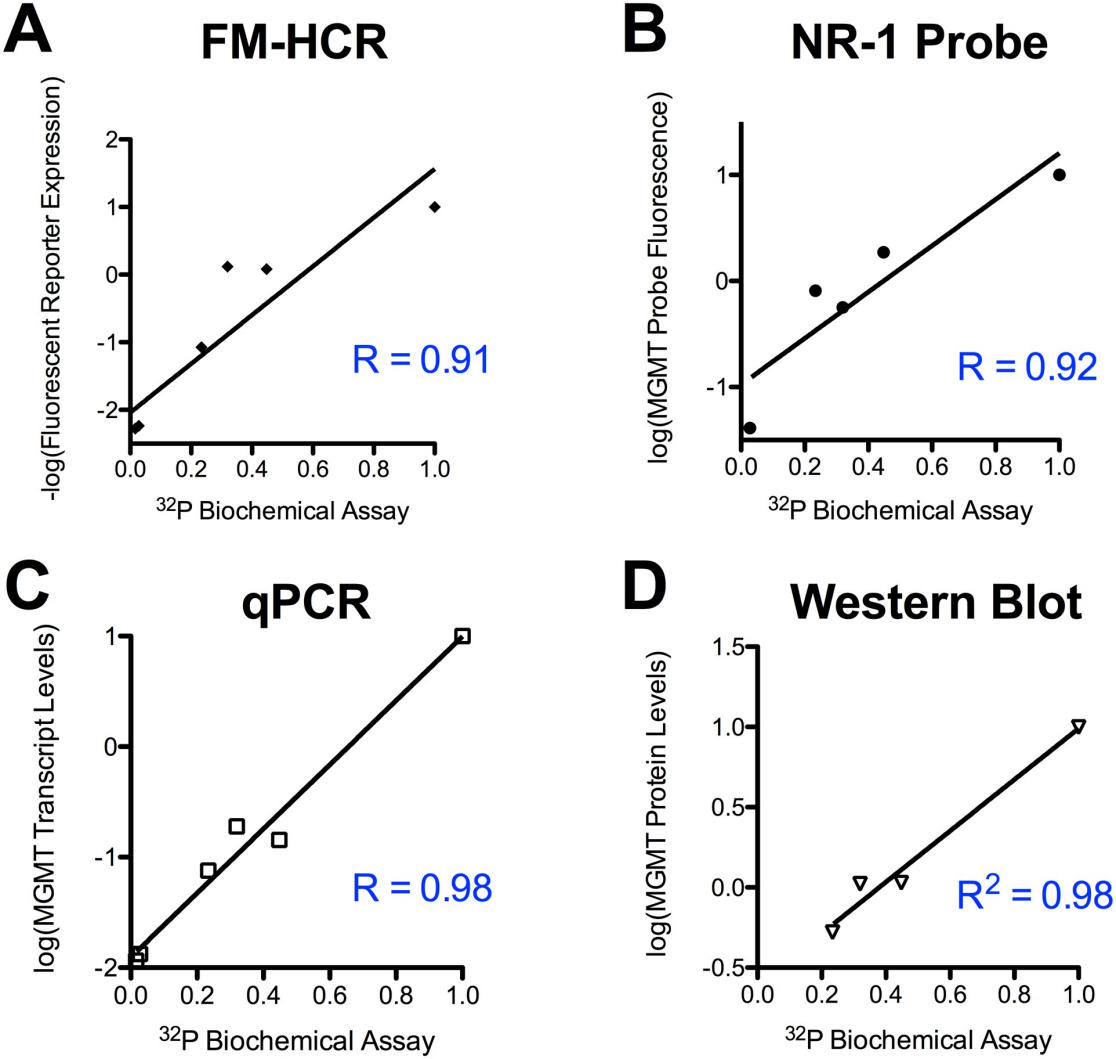
Comparison of four quantitative MGMT assays against the biochemical MGMT assay with radiolabeled oligonucleotides. All assays have been normalized to a control cell line (TK6+MGMT), which expresses a high level of MGMT. The Pearson correlation (R) to MGMT activity measured using biochemical assays with ^32^P-labeled oligonucleotide substrates is reported for each assay. Each data point represents one of the seven cell lines analyzed; fewer data points are reported for assays where some cell lines were below the limit of detection.

The gold standard radiolabeled oligonucleotide-based biochemical MGMT assay revealed an approximately 100-fold range of MGMT activity in the samples, with the following rank order established using the biochemical MGMT assay: TK6 < Coriell #5 < Coriell #4 < Coriell #12 < Coriell #14 < Coriell #16 < TK6+MGMT. The available quantitative methods for assessing MGMT status in human lymphoblastoid cell lines have been compared with this biochemical assay (Fig 2). All six methods yielded qualitatively similar estimates of MGMT activity in the seven cell lines, however each assay presents both unique technical demands and unique advantages (Table 1), detailed below.

**Table 1:** Sample requirements and capabilities of MGMT assays. Active time and total time were calculated for processing a single sample. Total time includes passive waiting time necessary for automated analytical processes and sample incubation. The estimates do not include time required to produce the oligonucleotides, fluorescent probes, antibodies and plasmids that are used.

	^32^P Biochemical	qPCR	Western Blot	MS-PCR	FM-HCR	NR-1 Probe
**Active Time [Total time], hours**	7 [15]	1.5 [5]	3 [13]	2 [5]	1.5 [20]	1.5 [3]
**Cells Required**^1^	10^7^−10^8^	10^3^	10^5^−10^6^	10^6^	10^6^	10^7^−10^8^
**Dynamic Range**^2^	62	62	4.3	NA	62	4.3
**Single set of conditions**	No	Yes	Yes	Yes	Yes	Yes
**Format**^3^	Lysate	Lysate	Lysate	Lysate	Intact	Lysate
**Materials Cost**^4^	$11	$5	$16	$10	$24	$0.06
**Direct measure of repair**	Yes	No	No	No	Yes	Yes

^1^Requirements refer to the approaches used here; a range is given for methods found to require more cells to lower MGMT levels.

^2^Dynamic range was calculated by dividing the activity (as measured using the biochemical assay) of the most active sample (TK6+MGMT) by the activity of the least active sample for which activity could be significantly distinguished from background.

^3^Intact refers to methods that can be carried out in live cells.

^4^Approximate cost of generating or purchasing materials needed to carry out each assay in triplicate using the approaches in methods.

### Oligonucleotide-based biochemical assays

*In vitro* biochemical MGMT activity assays can be regarded to be the “gold standard” for measuring MGMT activity because they directly measure the level of repair activity of MGMT protein using a chemically defined substrate with a radiolabel that permits detection of very low levels of repair activity. However, the assay has several technical drawbacks. Assay conditions must be optimized to determine the range of cell lysate protein concentrations that produce a linear response, which is cell-line dependent (Compare x-axes in panel C of S1 Fig). Furthermore, relative to assays measuring activity in the highly MGMT proficient cell line TK6+MGMT, an approximately 10-fold higher concentration of cell lysate protein was required to distinguish the very low level of MGMT activity in cell lines #4 and #5 from the undetectable activity in TK6 cells. Biochemical MGMT assays were the most time consuming of the four methods studied, requiring approximately 7 hours active laboratory time for analysis of up to 4 samples in parallel.

### FM-HCR

FM-HCR assays distinguished cells with low MGMT activity (Cell lines #4 and #5) from cells that lack MGMT activity (TK6), and thus exhibited the same dynamic range as the gold standard biochemical assay (A 62-fold range of activity comparing the least active sample, #5, to the most active sample, TK6). The sensitivity of FM-HCR is achieved in part because of the ability to detect individual cells harboring unrepaired DNA lesions that lead to transcriptional errors and fluorescent protein expression; whereas a minor subpopulation of repair deficient cells may be lost in ensemble measurements, they can be readily detected by FM-HCR. Together with qPCR and the fluorescent NR-1 probe assay (below), FM-HCR required the least amount of active laboratory time (1.5 hours) for analysis of up to 4 samples in parallel.

### Western blotting

As has been observed by others [28], MGMT protein levels estimated from Western blots correlated strongly with MGMT activity (R = 0.98, Fig 2D), however the low levels of MGMT in cell lines #4 and #5, detectable by the ^32^P-oligonucleotide-based biochemical assay, FM-HCR and qPCR, were below the limit of detection by western blotting. Western blots also required approximately twice the active laboratory time (3 hours for analysis of up to 4 samples in parallel) as the least labor-intensive assays.

### Transcript levels by qPCR

MGMT transcript levels measured by qPCR analysis, reported previously [17], correlate strongly (R = 0.98) with MGMT activity measured by the biochemical MGMT assay. Analysis by qPCR required approximately 1.5 hours for analysis of up to 4 samples in parallel.

### Fluorescent MGMT probe NR-1

MGMT activity as measured with the NR-1 probe correlated well with activity measured using the biochemical MGMT assay, however two cell lines (#4 and #5), which had the lowest activity as judged by the biochemical assay, were below the limit of detection. Analysis using the NR-1 probe required approximately 1.5 hours for analysis of up to 4 samples in parallel.

### Methylation specific PCR (MS-PCR)

Promoter methylation was detected, as expected, in TK6, previously shown to exhibit MGMT promoter methylation, and in TK6+MGMT, which is derived from TK6 and expresses MGMT constitutively under a CMV promoter [21]. Strikingly, MGMT promoter methylation was not detected in any of the lymphoblastoid cell lines, including those that express very low levels of MGMT, namely Coriell #5 and Coriell #4; this result highlights the potential for MS-PCR to inaccurately identify MGMT-deficient cells as MGMT proficient. Promoter methylation was also detected in two patient derived xenograft models of glioblastoma, GBM12_5199 and GBM12_3080, consistent with previous findings [25]. Notably, despite the robust MGMT promoter methylation observed in both cell lines (S3 Fig), GBM12_3080 expresses high levels of MGMT detectable by FM-HCR [18]. The conclusion that GBM12_3080 is proficient for MGMT is also supported by previous measurements of MGMT transcript levels and observed sensitization to TMZ upon treatment with the MGMT inhibitor *O*^6^-benzylguanine [29]. Analysis of MGMT promoter methylation by MS-PCR required approximately 2 hours for analysis of up to 4 samples in parallel.

## Discussion

The time-sensitive nature of cancer treatment as well as the serious side effects and risk of therapy-related cancers in patients treated with radiotherapy and chemotherapy have motivated a search for biomarkers that can predict whether specific therapies will work for individual patients [30]. The success of chemotherapy hinges upon the existence of a therapeutic window in which cancer cells can be killed without severe normal tissue toxicity. Acquired DNA repair defects drive genomic instability, a hallmark of cancer [31], and can sensitize cancer cells to chemotherapy [1]. Notably, MGMT defects occur in many cancers including glioblastoma [32], colorectal cancer [33], leukemia [34, 35], lymphoma [36], small cell lung cancer [36], breast cancer [37], pancreas cancer [38], and melanoma [39], and in some cases increases the effectiveness of therapeutic agents that generate DNA lesions that are MGMT substrates [40]. Thus, the ability to determine MGMT status accurately and efficiently in cancer cells could allow clinicians to tailor therapies to the needs of individual patients.

Although the biochemical MGMT assay is considered a gold standard because it provides a sensitive quantitative measure of functional MGMT levels in cell lysates, it was by far the most time consuming (7 hours active time for up to 4 samples processed in parallel, Table 1) due to the need for testing multiple conditions for establishing the linear range of the assay (S1 Fig). In addition, the biochemical assays required a radiolabeled oligonucleotide, and the largest number of cells (up to 10^8^) of the six assays used. These considerations render oligonucleotide-based assays too labor intensive for clinical use.

Three indirect assays, namely western blotting for MGMT protein levels, qPCR for MGMT transcript levels, and methylation specific PCR for MGMT promoter methylation status, were less labor intensive and required fewer cells. Indeed, promoter methylation assays, and to a lesser extent, immunohistochemistry, are currently used in the analysis of clinical samples; however, immunohistochemical approaches can fail to predict MGMT activity consistently [41], promoter methylation analysis can fail to predict transcript levels [25, 42], and MGMT transcript levels can fail to predict protein levels due to post-transcriptional regulation [43]. Furthermore, MGMT activity is affected by posttranslational modifications [44], and the repair protein is inactivated by its substrates following a single turnover [2]; these important contributions to MGMT activity cannot be detected by indirect assays. Thus, despite their promise for patient stratification in the context of alkylating chemotherapy, the challenges associated with existing MGMT assays has limited their potential for guiding therapy decisions.

The fluorescent probe NR-1, and FM-HCR assays overcome the problems associated with subjective histopathological scoring and the lack of correlation between promoter methylation and MGMT activity by providing a direct functional assessment of MGMT activity, without the need for radiolabeled probes or extensive sample processing. For example, methylation specific PCR indicated a total lack of MGMT promoter methylation in Coriell #4 (S3 Fig), which, in fact, expresses very low levels of MGMT according to the biochemical assay. FM-HCR assays detected the low level of MGMT activity present in Coriell #4, which was below the limit of detection by western blotting (S2 Fig), and required optimization of conditions for detection by the biochemical assay.

Two glioblastoma xenograft lines, GBM12_3080 and GBM12_5199, both exhibit MGMT promoter methylation (S3 Fig), but the FM-HCR assay correctly assigns GM12_3080 to be MGMT proficient [18], consistent with previous independent characterization [25]. One advantage of promoter methylation assays is that they are generally regarded to be specific for cancer cells, since the MGMT promoter is not methylated in normal cells. Although the fluorescence-based assays are not inherently specific for cancer cells, cell-type specificity could be achieved by carrying them out in conjunction with cell surface markers.

While both fluorescent reporter assays directly measure MGMT activity, their differing sample requirements and modes of detection endow them with complementary strengths. The FM-HCR assay must be used with intact cells, enabling detection of repair deficient subpopulations, and providing an integrated measure of MGMT activity over the course of 24 hours in cells, rather than a snapshot of MGMT activity at a single time point that the NR-1 probe provides. The estimated dynamic range for FM-HCR in the set of cell lines considered here (62) is larger than that of the NR-1 probe (4.3). Although the NR-1 probe failed to detect the very low levels of MGMT activity in extracts from cell lines #4 and #5, the probe did distinguish significant differences in activity among cell lines with higher MGMT activity (TK6+MGMT versus #12, #14, and #16), whereas FM-HCR did not (S2 Table). However FM-HCR requires transfection of live cells, and flow cytometric analysis, while the NR-1 probe can be used with cell lysates, and the fluorescent signal can be measured using a plate reader. Furthermore, the NR-1 probe can be used to measure repair kinetics in real time under varied conditions, such as in the presence of small molecule inhibitors. The NR-1 probe is also extremely cost-efficient because it is inexpensive to synthesize and requires only cell lysates and a plate reader for analysis.

Clinical translation will require studies in primary patient samples to determine how well these assays predict therapeutic outcomes assessed using standard measures such as the Response Evaluation Criteria in Solid Tumors (RECIST). For cell-based assays, optimization of tumor processing to maximize live cell content may be needed. Strategies for excluding signal from tumor stroma should be considered, for example using cell surface markers or immunomagnetic separation.

Limitations of existing assays have usually resulted in binary classification of tumor MGMT status, making it difficult to assess whether the distinction between tumors with low MGMT and those that are completely lacking MGMT is clinically important. However, available data are consistent with a continuous relationship between MGMT activity and temozolomide sensitivity [27]. Quantitative functional assays such as those presented herein will expand the potential for future studies aimed at resolving this question. Since FM-HCR reporters have been successfully transfected into both primary cells and cancer cells [17], and the NR-1 probe is amenable to any cells from which lysates can be derived [16], both approaches could potentially be used for studies of MGMT activity in cancerous and normal tissue.

## Conclusions

Many cancers exhibit alterations in MGMT activity that may be exploited when treating patients with alkylating chemotherapeutic agents, and MGMT activity in normal tissues may predict inter-individual differences in alkylating agent toxicity. However, there remains a critical need for accurate, functional assays that could be used to identify individuals with MGMT-deficient cancers. The recently developed FM-HCR assays and fluorescent NR-1 probe both overcome the problems associated with currently used indirect methods of measuring MGMT activity, and merit consideration as alternatives for use in pre-clinical studies and in clinical trials involving cancers where MGMT status may be associated with therapeutic outcomes.

## Supporting information

S1 Fig In vitro MGMT activity assay. A) Oligonucleotide digest assay for MGMT activity. Repair of a PstI cleavage blocking *O*^6^MeG DNA lesion results in an 8 nt 32P labeled fragment detectable by polyacrylamide gel electrophoresis. B) Polyacrylamide gel analysis of restriction digest products following treatment of 4 pmol of oligonucleotide with 10–400 μg of protein extracts from 7 lymphoblastoid cell lines for 30 minutes at 37 °C. C) Determination of linear range and calculation of MGMT activity. Slopes calculated from the data in these plots are reported in Fig 1, **Panel A**.
(PDF)

S2 Fig Representative immunoblot and quantitation of MGMT protein levels in 7 cell lines. Protein levels were below the limit of detection for Coriell #5, Coriell #4, and TK6.
(TIFF)

S3 Fig Gel electrophoretic analysis of methylation specific PCR products. Each lane shows PCR products obtained from amplification of bisulfite converted genomic DNA from the indicated cell lines with primers specific for unmethylated DNA (U), or methylated DNA (M).
(PDF)

S1 Table MGMT assay data and statistics. Units are as follows: ^**32**^**P Oligo**, fmoles cleaved oligonucleotide per microgram protein lysate; **NR-1**, Fluorescence signal, arbitrary units; **FM-HCR**, % Reporter Expression; **Western Blot**, MGMT protein levels as a percentage of actin protein levels; **qPCR**, MGMT transcript levels normalized to MGMT transcript levels in TK6+MGMT.
(DOCX)

S2 Table Pairwise statistical comparisons. Cell lines that were significantly different from one another for each MGMT assay are indicated with an asterisk “*”; those that were not significantly different are marked “ns”.
(DOCX)
