# Supplementary figures and images for: Fluorescent reporter assays provide direct, accurate, quantitative measurements of MGMT status in human cells
Source: PLoS One. 2019 Feb 27;14(2):e0208341. doi: 10.1371/journal.pone.0208341 (PMC6392231; doi:10.1371/journal.pone.0208341)

**A**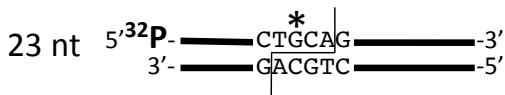

Repair incubation  
↓  
PstI digest

↓  
Denaturing PAGE

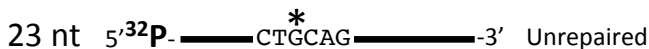

OR

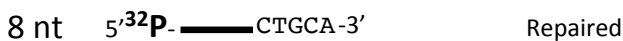**B**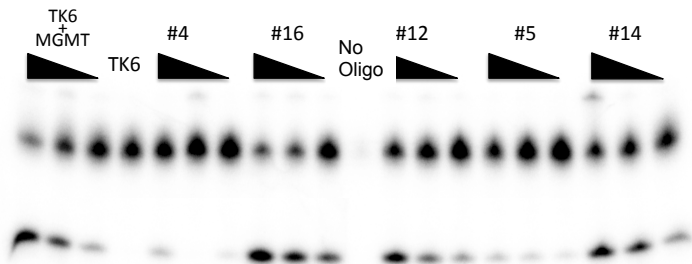**C**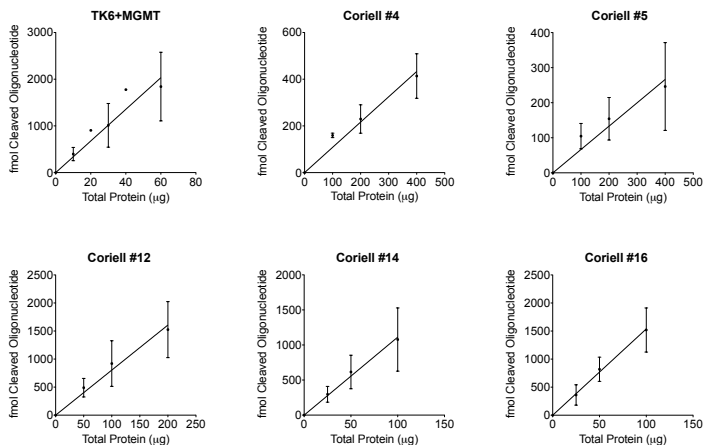

Supplement: S1 Fig — A) Oligonucleotide digest assay for MGMT activity. Repair of a PstI cleavage blocking O6MeG DNA lesion results in an 8 nt 32P labeled fragment detectable by polyacrylamide gel electrophoresis. B) Polyacrylamide gel analysis of restriction digest products following treatment of 4 pmol of oligonucleotide with 10–400 μg of protein extracts from 7 lymphoblastoid cell lines for 30 minutes at 37 °C. C) Determination of linear range and calculation of MGMT activity. Slopes calculated from the data in these plots are reported in Fig 1, Panel A. (PDF) [file pone.0208341.s001.pdf]

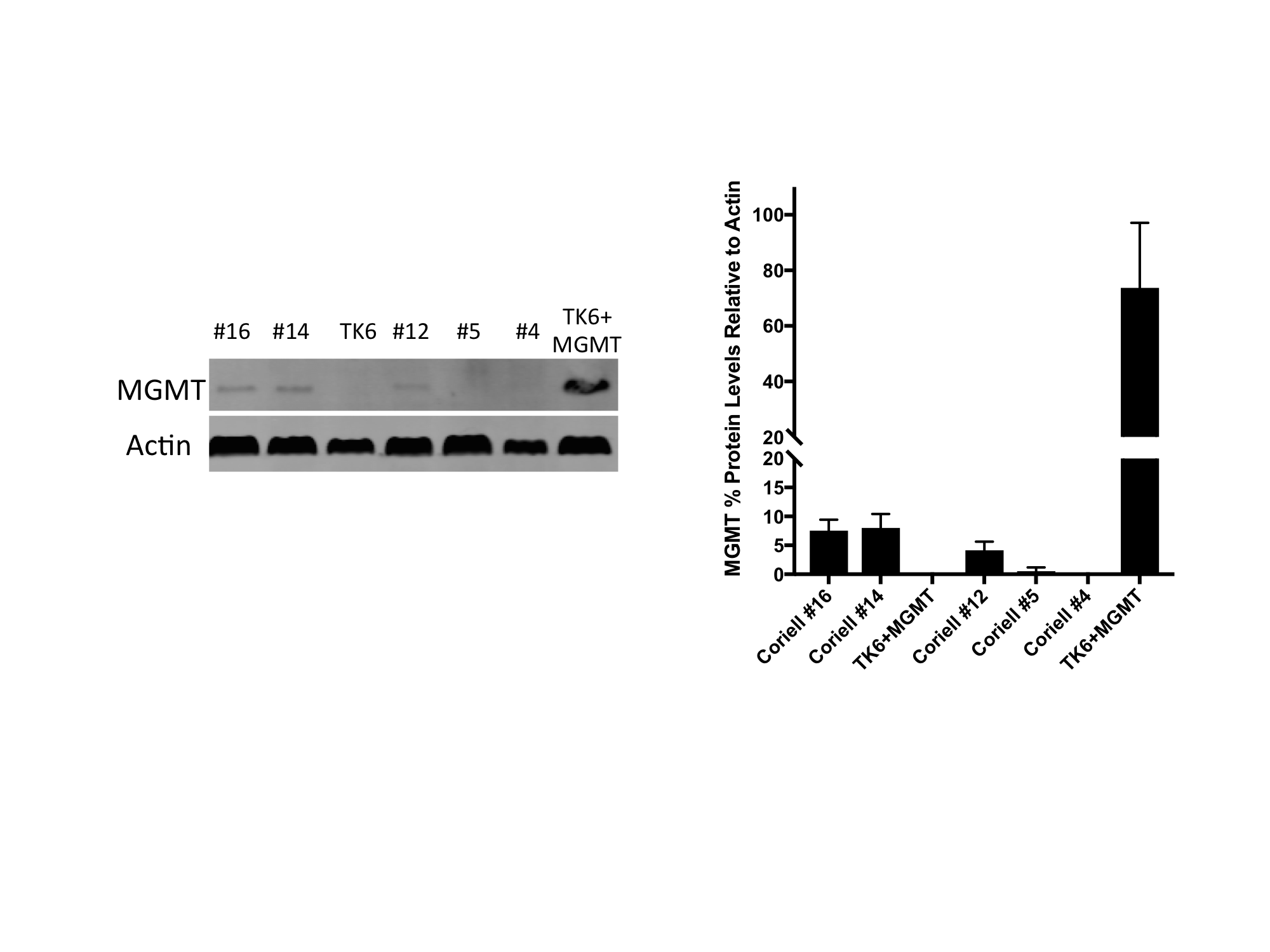

Supplement: S2 Fig — Protein levels were below the limit of detection for Coriell #5, Coriell #4, and TK6. (TIFF) [file pone.0208341.s002.tiff]

# Coriell

**TK6**

**#4**

**#5**

**#12**

**#14**

**#16**

**3080**

**5199**

**U M**

100bp ➔

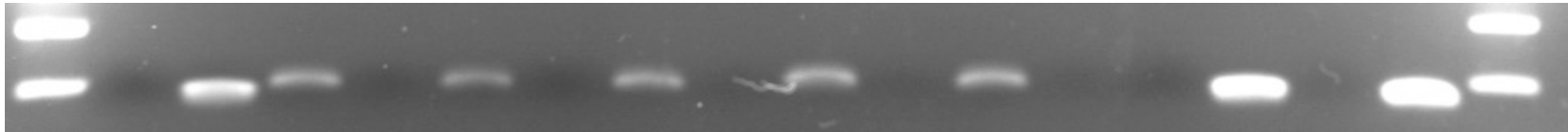

Supplement: S3 Fig — Each lane shows PCR products obtained from amplification of bisulfite converted genomic DNA from the indicated cell lines with primers specific for unmethylated DNA (U), or methylated DNA (M). (PDF) [file pone.0208341.s003.pdf]
