# Supplementary material for: Fluorescent reporter assays provide direct, accurate, quantitative measurements of MGMT status in human cells
Source: PLoS One. 2019 Feb 27;14(2):e0208341. doi: 10.1371/journal.pone.0208341 (PMC6392231; doi:10.1371/journal.pone.0208341)
Supplement: S1 Table — Units are as follows: 32P Oligo, fmoles cleaved oligonucleotide per microgram protein lysate; NR-1, Fluorescence signal, arbitrary units; FM-HCR, % Reporter Expression; Western Blot, MGMT protein levels as a percentage of actin protein levels; qPCR, MGMT transcript levels normalized to MGMT transcript levels in TK6+MGMT. (DOCX) [file pone.0208341.s004.docx]

|  | **^32^P Oligo** | | **NR-1** | | **FM-HCR** | | **Western** | | **qPCR** | |
| --- | --- | --- | --- | --- | --- | --- | --- | --- | --- | --- |
|  | **Avg** | **Error** | **Avg** | **Error** | **Avg** | **Error** | **Avg** | **Error** | **Avg** | **Error** |
| TK6 | 0.09 | 0.02 | - | - | 50.1 | 5.4 | - | - | 8.0E-06 | 1.0E-06 |
| #5 | 0.51 | 0.14 | - | - | 28.8 | 1.3 | - | - | 1.2E-03 | 1.6E-05 |
| #4 | 0.89 | 0.07 | 0.3 | 4.8 | 26.2 | 4.4 | - | - | 1.3E-03 | 5.2E-04 |
| #12 | 7.4 | 1.1 | 6.2 | 2.7 | 1.8 | 0.84 | 3.8 | 1.1 | 7.6E-03 | 8.2E-04 |
| #14 | 10.5 | 1.7 | 4.4 | 1.9 | 0.12 | 0.22 | 7.6 | 2.7 | 1.9E-02 | 8.1E-04 |
| #16 | 15.0 | 1.6 | 14.4 | 7.5 | 0.13 | 0.06 | 7.7 | 1.5 | 1.4E-02 | 1.7E-03 |
| TK6+MGMT | 31.8 | 4.3 | 77.2 | 13.3 | 0.02 | 0.01 | 72.3 | 25.2 | 1.0E+00 | - |
