# Supplementary material for: Fluorescent reporter assays provide direct, accurate, quantitative measurements of MGMT status in human cells
Source: PLoS One. 2019 Feb 27;14(2):e0208341. doi: 10.1371/journal.pone.0208341 (PMC6392231; doi:10.1371/journal.pone.0208341)
Supplement: S2 Table — Cell lines that were significantly different from one another for each MGMT assay are indicated with an asterisk “*”; those that were not significantly different are marked “ns”. (DOCX) [file pone.0208341.s005.docx]

| **FM-HCR** | | | | | | | | | | | | | | | | | | | |
| --- | --- | --- | --- | --- | --- | --- | --- | --- | --- | --- | --- | --- | --- | --- | --- | --- | --- | --- | --- |
|  | **TK6+MGMT** | | **#16** | **#14** | **#12** | | | **#4** | | | | **#5** | | | |  |  |  |  |
| **TK6** | * | | * | * | * | | | * | | | | * | | | |  |  |  |  |
| **#5** | * | | * | * | * | | | ns | | | |  | | | |  |  |  |  |
| **#4** | * | | * | * | * | | |  | |  | | | |  |  |  |  |  |  |
| **#12** | ns | | ns | ns |  | | |  | |  | | | |  |  |  |  |  |  |
| **#14** | ns | | ns |  |  | | |  | |  | | | |  |  |  |  |  |  |
| **#16** | ns | |  |  |  | | |  | |  | | | |  |  |  |  |  |  |
|  | |  | | | | |  | | | |  | | | |  | | |  |  |
| **qPCR** | | | | | | | | | | | | | | | | | | | |
|  | **TK6+MGMT** | | **#16** | **#14** | **#12** | | | **#4** | | | | **#5** | | | |  |  |  |  |
| **TK6** | * | | * | * | * | | | ns | | | | ns | | | |  |  |  |  |
| **#5** | * | | * | * | * | | | ns | | | |  | | | |  |  |  |  |
| **#4** | * | | * | * | * | | |  | |  | | | |  |  |  |  |  |  |
| **#12** | * | | * | * |  | | |  | |  | | | |  |  |  |  |  |  |
| **#14** | * | | * |  |  | | |  | |  | | | |  |  |  |  |  |  |
| **#16** | * | |  |  |  | | |  | |  | | | |  |  |  |  |  |  |
|  |  | | | | |  | | |  | | | |  | | | |  |  |  |
| **Western** | | | | | | | | | | | | | | | | | | | |
|  | **TK6+MGMT** | | **#16** | **#14** | **#12** | | | **#4** | | | | **#5** | | | |  |  |  |  |
| **TK6** | * | | ns | ns | ns | | | ns | | | | ns | | | |  |  |  |  |
| **#5** | * | | ns | ns | ns | | | ns | | | |  | | | |  |  |  |  |
| **#4** | * | | ns | ns | ns | | |  | | | |  | | | |  |  |  |  |
| **#12** | * | | ns | ns |  | | |  | |  | | | |  |  |  |  |  |  |
| **#14** | * | | ns |  |  | | |  | |  | | | |  |  |  |  |  |  |
| **#16** | * | |  |  |  | | |  | |  | | | |  |  |  |  |  |  |
|  | |  | | | | |  | | | |  | | | |  | | |  |  |
| **NR-1** | | | | | | | | | | | | | | | | | | | |
|  | **TK6+MGMT** | | **#16** | **#14** | **#12** | | | **#4** | | | | **#5** | | | |  |  |  |  |
| **TK6** | * | | ns | ns | ns | | | ns | | | | ns | | | |  |  |  |  |
| **#5** | * | | ns | ns | ns | | | ns | | | |  | | | |  |  |  |  |
| **#4** | * | | ns | ns | ns | | |  | |  | | | |  |  |  |  |  |  |
| **#12** | * | | ns | ns |  | | |  | |  | | | |  |  |  |  |  |  |
| **#14** | * | | ns |  |  | | |  | |  | | | |  |  |  |  |  |  |
| **#16** | * | |  |  |  | | |  | |  | | | |  |  |  |  |  |  |
|  | |  | | | | |  | | | |  | | | |  | | |  |  |
| **Oligo Assay** | | | | | | | | | | | | | | | | | | | |
|  | **TK6+MGMT** | | **#16** | **#14** | **#12** | | | **#4** | | | | **#5** | | | |  |  |  |  |
| **TK6** | * | | * | * | * | | | * | | | | ns | | | |  |  |  |  |
| **#5** | * | | * | * | * | | | ns | | | |  | | | |  |  |  |  |
| **#4** | * | | * | * | * | | |  | |  | | | |  |  |  |  |  |  |
| **#12** | * | | ns | ns |  | | |  | |  | | | |  |  |  |  |  |  |
| **#14** | * | | ns |  |  | | |  | |  | | | |  |  |  |  |  |  |
| **#16** | * | |  |  |  | | |  | |  | | | |  |  |  |  |  |  |
